# 2221. Comparison of Vancomycin with Either Piperacillin/Tazobactam or Cefepime for the Incidence of Acute Kidney Injury in a Patient Population with a High Prevalence of Preexisting Kidney Dysfunction

**DOI:** 10.1093/ofid/ofad500.1843

**Published:** 2023-11-27

**Authors:** Ryan Moran, Jacklyn Harris, Jessica Kolkmeyer

**Affiliations:** HSHS St. John's Hospital, Palatine, Illinois; BJC Christian Hospital, St. Louis, Missouri; BJC Christian Hospital, St. Louis, Missouri

## Abstract

**Background:**

Frequently used agents for empiric antibiotic therapy include vancomycin, cefepime, and piperacillin/tazobactam (PTZ). Documentation exists to suggest the combination of vancomycin and PTZ increases the risk of acute kidney injury (AKI) more than either alone. This interaction has not been specifically studied in a population with a high prevalence of preexisting kidney dysfunction. The purpose of this study is to investigate if the combination of vancomycin and PTZ increases the incidence of AKI compared to vancomycin with cefepime in this population.

**Methods:**

This retrospective study compares hospitalized patients receiving vancomycin with either cefepime or PTZ. Inclusion criteria were age ≥ 18 and ≥ 72 hours concomitant receipt of vancomycin with either PTZ or cefepime. Exclusion criteria were AKI or dialysis prior to antibiotic initiation, receipt of both cefepime and PTZ, and being COVID-19 positive. Patients were assigned to one of the groups based on the antibiotics they received.

The two co-primary outcomes were the incidence of AKI in the entire study population and specifically in those with preexisting chronic kidney disease (CKD). Secondary outcomes include duration of hospitalization, duration of antibiotic therapy, mortality, severity of AKI, and incidence of dialysis initiation.

**Results:**

Of 470 candidates screened, 276 were included in the trial with 164 and 112 in the cefepime and PTZ groups, respectively. Of these patients, 39% had CKD (185/470). Groups were evenly distributed concerning baseline characteristics except the cefepime group was significantly older by an average of 7.3 years (p < 0.001). The primary outcome in the total study population occurred in 17.1% in the cefepime group and 39.3% in the PTZ group (p < 0.001). In the CKD population, the PTZ group had a higher rate of AKI at 48.2% compared to 38% of cefepime (p = 0.389). There were no significant differences in any secondary outcomes.

**Conclusion:**

These results suggest the combination of vancomycin and PTZ increases the risk of AKI. The study was unable to show a significant difference for those with preexisting CKD. It is unknown whether this is related to the presence of CKD or the small sample size. Additional research is needed to further evaluate vancomycin with PTZ in certain patient populations.

**Disclosures:**

**All Authors**: No reported disclosures

